# Latitudinal Variation in Circadian Rhythmicity in *Nasonia vitripennis*

**DOI:** 10.3390/bs9110115

**Published:** 2019-11-15

**Authors:** Silvia Paolucci, Elena Dalla Benetta, Lucia Salis, David Doležel, Louis van de Zande, Leo W. Beukeboom

**Affiliations:** 1 Groningen Institute for Evolutionary Life Sciences, University of Groningen, 9712 CP Groningen, The Netherlands; silvia.paolucci81@gmail.com (S.P.); lucia.salis@gmail.com (L.S.); dolezel@entu.cas.cz (D.D.); l.w.beukeboom@rug.nl (L.W.B.); 2 Institute of Entomology, Biology Center of the Czech Academy of Sciences, 370 05 Ceske Budejovice, Czech Republic; louis.van.de.zande@rug.nl

**Keywords:** circadian clock, *Nasonia vitripennis*, latitudinal cline, free running period, *period*

## Abstract

Many physiological processes of living organisms show circadian rhythms, governed by an endogenous clock. This clock has a genetic basis and is entrained by external cues, such as light and temperature. Other physiological processes exhibit seasonal rhythms, that are also responsive to light and temperature. We previously reported a natural latitudinal cline of photoperiodic diapause induction in the parasitic wasp *Nasonia vitripennis* in Europe and a correlated haplotype frequency for the circadian clock gene *period* (*per*). To evaluate if this correlation is reflected in circadian behaviour, we investigated the circadian locomotor activity of seven populations from the cline. We found that the proportion of rhythmic males was higher than females in constant darkness, and that mating decreased rhythmicity of both sexes. Only for virgin females, the free running period (*τ*) increased weakly with latitude. Wasps from the most southern locality had an overall shorter free running rhythm and earlier onset, peak, and offset of activity during the 24 h period, than wasps from the northernmost locality. We evaluated this variation in rhythmicity as a function of *period* haplotype frequencies in the populations and discussed its functional significance in the context of local adaptation.

## 1. Introduction

The daily rotation of the Earth around its axis causes oscillating photoperiods that have led to the evolution of a large variety of activity patterns of organisms. Many behavioural and physiological activities, like mating, feeding, and resting, show distinct oscillating rhythms with a peak of activity at a specific moment during the light–dark (LD) cycle. These are driven by an endogenous clock that is reset daily (entrained) by the prevailing LD cycles and runs with an intrinsic period of approximately 24 h in constant darkness (DD) [1]. The length of this endogenous rhythm is called the free running period (*τ*). 

Day length (photoperiod) also oscillates seasonally and is an important cue for season-dependent behaviours, like migration in birds, hibernation in mammals, and diapause in insects (reviewed in [2]). Additionally, daily photoperiods depend on latitude, being almost constant near the equator and increasing in yearly variation towards higher latitudes. Hence, depending on latitude, organisms will experience different photoperiods over the year. Given the sensitivity of the circadian clock to light–dark fluctuations, it is conceivable that it also plays a role in seasonal rhythmicity [3]. There is accumulating evidence for a role of the circadian clock genes in photoperiodism in many species [4,5,6,7,8], and several studies have shown that seasonal responses differ geographically as a result of variation in photoperiodic conditions [9]. Nevertheless, it is still unclear whether the observed natural variation in photoperiodic response is controlled by specific circadian clock properties as a whole, such as the pace and the phase of the endogenous clock, or by pleiotropy of individual clock genes [8,10].

Larval diapause of the parasitoid *Nasonia vitripennis* is maternally induced following a certain number of days (switch point) at a given critical photoperiod (CPP) and shows a robust clinal photoperiodic response [11,12]. Apparently, a clock mechanism is responsible for the timing and counting of the light–dark cycles necessary for proper starting of the photoperiodic response [12]. Under long photoperiods, the switch point to start inducing diapause occurs late in life or not at all [13]. Interestingly, haplotype frequency distribution of the circadian clock gene *period (per)* follows the observed cline in photoperiodic diapause induction [11,14]. 

To investigate if the observed correlation of *per* haplotypes with seasonal response is reflected in natural variation in circadian activities, that are known to be regulated by the gene *period* [8], we analysed seven populations collected along a European cline from Corsica to northern Finland. We first tested variation in the free running period *τ* and then analysed the timing and level of locomotor activity for the most southern and most northern populations. Our data indicate that activity timing and average free-running rhythm differ between southern and northern lines of *N. vitripennis*, suggesting a latitude-dependent effect on the circadian clock, consistent with clinal *per* haplotypes.

## 2. Materials and Methods

### 2.1. Experimental Lines

To study variation in locomotor activity in *Nasonia vitripennis*, we used isofemale lines established from natural field collected populations [11]. These lines originated from seven European sampling locations (OUL (Finland, Oulu): 65°3′40.16″ N, 25°31′40.80″ E; TUR (Finland, Turku): 61°15′40.53″ N, 22°13′23.96″ E; LAT (Latvia): 56°51′22.56″ N, 25°12′1.38″ E; HAM (Germany, Hamburg): 53°36′23.62″ N, 10°10′17.74″ E; SCH (Germany, Schlüchtern): 50°19′56.10″ N, 9°30′47.00″ E; SWI (Switzerland): 46°44′9.14″ N, 7°6′57.34″ E; COR (France, Corsica): 42°22′40.80″ N, 8°44′ 52.80″ E). Wasps were maintained on *Calliphora* spp. pupae as hosts in mass culture vials under LD 18:06, 20 ± 1 °C, to minimise diapause induction. For establishing free running periods under constant darkness (DD), 17–25 isofemale lines from each location and 4–8 individuals from each isofemale line were used (797 females and 715 males). As some individuals died before all data were collected, only 1072 individuals could be used for locomotor activity analysis: 548 females (163 virgin and 385 mated) and 544 males (122 virgin and 402 mated). 

### 2.2. Locomotor Activity Recording 

To quantify animal movement over time, individuals were collected one day after emergence (mated group) or collected as pupae and allowed to develop into adults at room temperature (virgin group) and kept either at LD 16:08 or LD 08:16 based on experimental group. Adults were then individually transferred, without anaesthetization, to glass tubes (diameter 5 mm × height 70 mm) that were half filled with an agar gel containing 30% sugar. Trikinetics *Drosophila* activity monitors 2 (DAM2) (www.trikinetics.com) were used for activity registration, with 32 wasps per monitor. All assays were performed in light-tight boxes in temperature-controlled environmental chambers at 20 °C and 50% humidity. The light source in the box consisted of a white light with a maximum light intensity of about 200 lum/sqf. The Trikinetics system monitored how many times per minute each individual wasp interrupted an infrared light beam that passed through the center of the glass tube. To determine free-running period under constant darkness (DD), wasps were first entrained under LD16:8 for 4 days and then subjected to 10 days of DD. To compare daily activity profile under long and short photoperiods, adult wasps were recorded for 10 days in either LD 16:08 or LD 08:16 regime.

### 2.3. Data Analysis and Statistics

The raw locomotor activity data were first visualised with the program ActogramJ [15]; available at http://actogramj.neurofly.de. Double-plot actograms obtained with this software were eye inspected and dead animals were omitted from further analysis. Under constant darkness (DD) it was possible to measure the period of activity (*τ*) with periodogram analysis available in ActogramJ, which incorporated the chi-square test [16]. Linear mixed effect models were used to test the effect of location, latitude, sex, and mating status on the percentage of rhythmic individuals and on the length of the free running period *tau*, with the isofemale line nested into location as random effect (package *nlme*). The assumptions for parametric statistics were tested through diagnostic QQ plots. Only rhythmic individuals were included in these tests. All statistical analyses were performed with R statistical software (version 3.4.1, R Development Core Team 2012). 

Under LD conditions the average activity was calculated as described by [17]. The first four days of entrainment were excluded from the analysis. To determine the onset and offset of activity on each day, data per wasp have to be plotted as bar diagrams with each bar representing the sum of activity within 20 min. The onset represents the first time when activity started to rise consecutively, whereas the offset is when activity reached the level which was stable during the night phase. To determine the timing of the peaks, the data were smoothed by a moving average of 30 min. Through this process, randomly occurring spikes were reduced and the real maximum of the activity could be determined. The average phase of the onset, peak, and offset, represented in Zeitgeber time (ZT, where ZT 0 represents the time when the light turned on), was compared between different lines and treatments. 

## 3. Results

### 3.1. Rhythmicity and Free Running Periods (τ)

**Figure 1:**
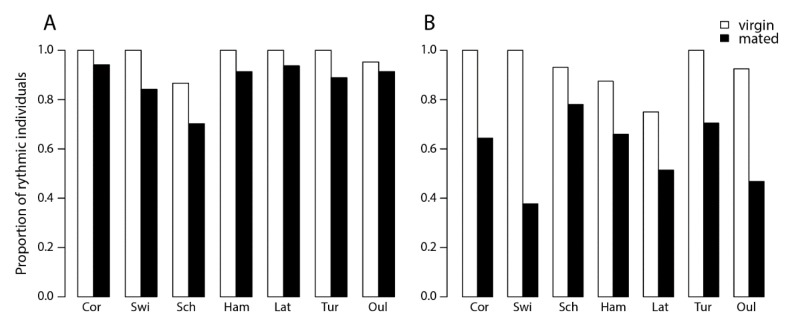
Proportion of rhythmic *Nasonia vitripennis* individuals in populations originating from seven locations in Europe in (**A**) males and (**B**) females. Locations along the x-axis are arranged from lower to higher latitude, see text for locality details. Generalized linear model (GLM) statistical analysis: effect of sex: χ^2^ = 65.71, *p* < 0.01; effect of location χ^2^ = 33.00, *p* < 0.01, in both females and males (effect of location: for females χ^2^ = 13.28, *p* < 0.05; for males χ^2^ = 13.46, *p* < 0.05); effect of latitude: χ^2^ = 1.77, *p* = 0.18, in both females and males (effect of latitude: for females χ^2^ = 1.65, *p* = 0.19; for males χ^2^= 0.16, *p* = 0.68); effect of mating status within females: χ^2^ = 50.25, *p* < 0.01; effect of mating status within males: χ^2^ = 12.18, *p* < 0.01; effect of latitude: for virgin individuals χ^2^ = 2.22, *p* = 0.13 and for mated individuals χ^2^ = 1.60, *p* = 0.20. (OUL = Finland, Oulu; TUR = Finland, Turku; LAT = Latvia; HAM = Germany, Hamburg; SCH = Germany, Schlüchtern; SWI = Switzerland; COR = France, Corsica.

**Figure 2:**
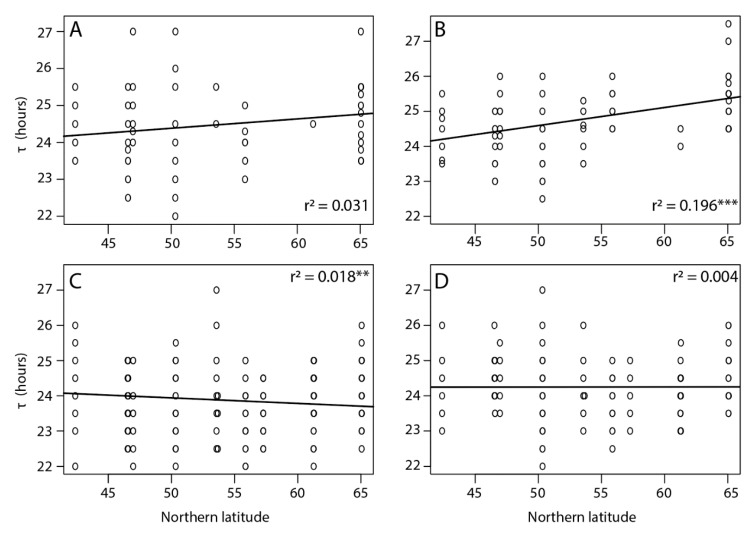
Free running period (τ) of (**A**) virgin males, (**B**) virgin females, (**C**) mated males, and (**D**) mated females in *Nasonia vitripennis* populations collected along a latitudinal gradient in Europe. Asterisks indicate a significant effect of location along the cline (*** *p* < 0.001 and ** *p* < 0.05, linear mixed effect model). Effect of sex: *LRT* = 2.22, *p* = 0.13; effect of mating status: *LRT* = 44.32, *p* < 0.01; effect of location: *LRT* = 38.38, *p* < 0.01; effect of location for virgin females *LRT* = 14.56, *p* < 0.05 and for mated males *LRT* = 13.02, *p* < 0.05.

The proportion of rhythmic individuals within a population ranged from 75% (SWI) to 84% (COR). When considering the complete dataset, males were more rhythmic (90%) than females (68%) (*p* < 0.001), with small but significant differences between locations *(p* < 0.01). There was no correlation with latitude (*p* = 0.18), either for the overall data or for the sexes separately (Figure 1). Virgin individuals were more rhythmic than mated individuals in both sexes *(p* < 0.01), but no latitudinal cline of rhythmicity was detected when mating status was taken into account (*p* = 0.20) (Figure 2). These results indicate variation between populations, sexes, and mating status in proportions of rhythmic individuals, but this variation did not follow a geographical cline.

Among all tested individuals there was a large variation in *τ*, which ranged from 22 h in the southern Corsica lines to 27.5 h in the northern Oulu lines. Overall, the free running period was shorter for individuals of southern latitudes and increased towards the north with a shallow but significant latitudinal cline in *τ* detected only for virgin females (*p* < 0.001) (Figure 2). The average *τ* for virgin COR females was 24.6 ± 0.12 h and for OUL 25.42 ± 0.11 h, corresponding to a difference of 49.2 min. Additionally, longer τ were observed in females compared to males (24.39 ± 0.04 and 23.97 ± 0.04 h, respectively, *p* < 0.01) and in virgin individuals compared to mated individuals (24.49 ± 0.05 and 24.00 ± 0.03 h, respectively, *p* < 0.01).

### 3.2. Activity Timing 

**Figure 3:**
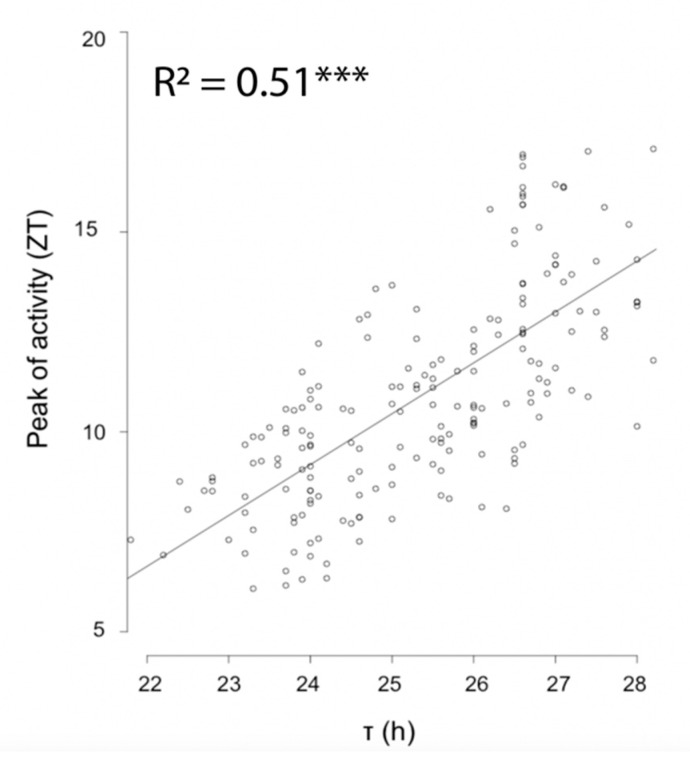
Correlation between peak of activity and free running period. Free running period (τ) of southern and northern *Nasonia vitripennis*. Asterisks indicate a significant effect of activity timing on free running period (*** *p* < 2e-16, linear mixed effect model).

**Figure 4:**
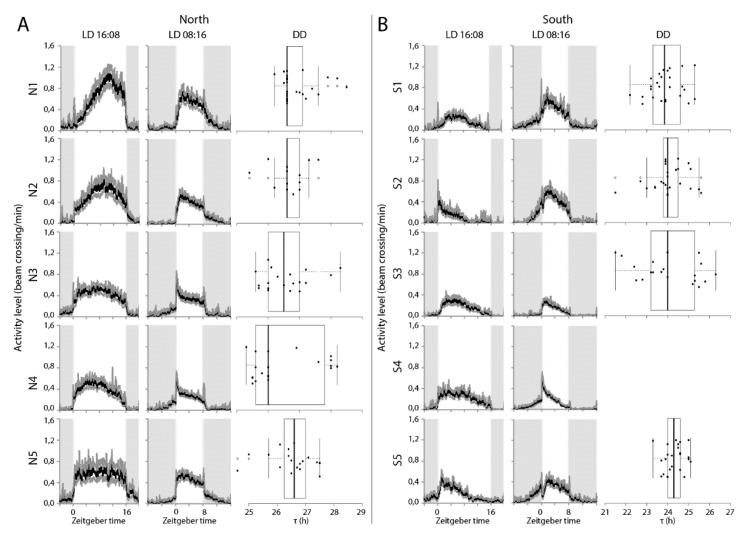
(**A**) Locomotor activity profiles for isogenic lines derived from Oulu (65 °N) (N1, N2, N3, N4, N5) and (**B**) from Corsica (45 °N) (S1, S2, S3, S4, S5) at long (LD16:08) and short (LD 08:16) day regimes. Grey shading indicates the night phase and white shading indicates the day phase. Zeitgeber time is indicated along the X-axis and ZT0 represents the time of light turn-on. Activity was calculated as average of bin crosses/minute of 25–32 individuals each over 24 h periods. Box plots represent the free running period (τ) in constant darkness (DD). Box plots depict the median (thick horizontal line within the box), the 25th and 75th percentiles (box margins) and the 1.5 interquartile range (thin horizontal line). Note that Line S4 is not rhythmic under DD. Note different scale on x-axis for A and B panels.

Virgin females from five isogenic lines established from populations of the two extremes of the sampling range were exposed to a light-dark regime of either LD16:08 or LD08:16h for four days as well as under free-running conditions. Examination of activity phases revealed a strong correlation between free-running period length and phase of peak activity (Figure 3, *p* < 2^−16^). Under LD16:08, both southern and northern wasps displayed a unimodal activity pattern (Figure 4), but with significant differences in the timing of the onset, peak, and offset of activity (Tables S1 and S2). Southern wasps started activity on average around ZT 0, which is about two hours earlier than northern wasps (Figure 4, Tables S1 and S2). Southern wasps displayed maximum activity around ZT 5, while northern wasps peaked at ZT 8 (Figure 4, Tables S1 and S2). Offset of activity was around ZT 13 and ZT 16 for southern and northern wasps, respectively (Figure 4, Tables S1 and S2). Thus, southern wasps were more active in the first half of the light period and northern wasps towards the end of the day. 

Southern wasps also started their activity earlier than northern wasps under the shorter photoperiod LD08:16 (Figure 4, Tables S1 and S3). Onset of activity occurred when the light was still off, around ZT 21.5. Northern wasps became active at ZT 0 (Figure 4, Tables S1 and S2). The peaks of activity differed by about one-and-half hours, at ZT 2.5 and ZT 4 for southern and northern wasps, respectively (Figure 4, Tables S1 and S3). Offset of activity was at ZT 8 for southern wasps. Northern wasps prolonged activity for more than two hours into darkness, until ZT 10.5, on average (Figure 4, Tables S1 and S3). Thus, under both light regimes there was a difference in phase of activity between southern and northern wasps. The consequence is that in the short photoperiod, southern wasps start activity in the dark and finish in the light phase, whereas northern wasps start activity at the beginning of the light phase and continue in the dark. 

In agreement with the results obtained for the isofemale lines, the southern and northern isogenic lines differed in τ under constant conditions (Figure 4, Table S1). The average free-running period of southern wasps was 24.3 ± 0.1 h, which differed significantly from the longer τ of 26.7 ± 0.1 h of the northern ones (*p* < 0.001). 

## 4. Discussion

*Nasonia vitripennis* has a broad distribution and it is thus expected to exhibit natural variation in biological rhythms [9]. Latitudinal cline variation in diapause induction (seasonal response), correlating with the clock gene *per,* has already been reported by [11,14]. Here, we describe natural variation for several properties of circadian locomotor activity of *N. vitripennis*. We observed significant differences between females and males: males were more rhythmic than females and had shorter free-running periods (*τ*), and this difference was more apparent in mated individuals. Similar differences between sexes were observed by [18] and in the laboratory strain *N. vitripennis* AsymC [19]. Interestingly, virgin individuals of both sexes were highly rhythmic in constant darkness and most females lost their internal rhythmicity after mating. Similar effects of mating status on rhythmicity were found in the ant species *Camponotus compressus,* in which ovipositing queens exhibited arrhythmic locomotor activity during the egg laying phase and restored rhythmicity afterwards [20]. In addition, we found a significant interaction between locality and mating status on the proportion of rhythmic individuals that might reflect standing genetic variation for rhythmic behaviour within and among populations. It is possible that environmental factors affect the rhythmic locomotor activity, as recently shown for the northern fly species *Drosophila montana*, in which the proportion of rhythmic individuals was higher at a lower temperature [21]. More studies investigating the influence of environmental factors (e.g., temperature and light intensity) on circadian locomotor activity in males and females in various species could potentially reveal differential selection pressures for stability of the circadian clock under different conditions. 

We also found differences in the free running period (*τ*) between sexes and locations. Towards the south, wasps had a faster clock, with *τ* close to 24 h, whereas wasps from northern latitudes had a slower clock with *τ* longer than 24 h. In virgin females, we observed a weak, but significant, latitudinal cline for τ, increasing towards higher latitudes. The presence of a positive latitudinal cline from south to north in DD rhythm was previously reported for *Drosophila* species [22,23], but only few studies have addressed variation in free running rhythms within a species. For example, in the model plant *Arabidopsis thaliana* the free running period under DD increases towards a northern latitude, and correlates with clinal variation in seasonal flowering time, regulated by photoperiodic cycles [24]. In insects, similar results (i.e., longer *τ* towards northern latitude) were reported from the mosquito *Culex pipiens* [25] and the linden bug *Pyrrhocoris apterus* [26]. This suggests that the latitudinal differences in free running period are the result of a selection of traits that enable local adaptation. One possibility is selection for phase of activity, in which a faster clock corresponds to an earlier activity phase and a slower clock is associated with later activity phase. 

The period of the circadian clock of *Nasonia* females may reflect the timing of locomotor activity under LD conditions. Indeed, we observed a positive correlation between the activity phase and free running rhythm of the wasps (i.e., wasps with shorter *τ* had earlier activity peak). Southern and northern wasps displayed profound differences in their daily locomotor activity. Southern wasps were mainly active in the morning, with an increase in activity before the light turned on during the short photoperiod, whereas northern ones presented a unimodal evening activity, with a prolonged evening peak at the shorter photoperiod. This shifted activity pattern between southern and northern wasps can reflect local adaptation. In the south, temperatures are known to become high in the middle to late afternoon, and shifting the activity to the coolest part of the day (the morning) might be a response of insects that live in a hot environment [22,23]. In contrast, species that live at higher latitudes have to cope with lower temperatures and longer photoperiods [27]. Northern *Nasonia* lines have a reduced morning activity with their activity peak in the second part of the day when temperatures are higher. Similar differences in activity patterns between southern and northern populations have been reported for *Drosophila*, albeit between *Drosophila* species rather than populations within species [23,24,25,26,27]. However, the overall activity profile of *N. vitripennis* was rather broad compared to the more precisely timed behaviour of *Drosophila melanogaster*, possibly reflecting a stronger selection on activity phase in *D. melanogaster* than *N. vitripennis*. On the other hand, *Nasonia* exhibited a stronger photoperiodic response [13]. 

The association between *per* polymorphisms and circadian phase of activity has also been observed in *Drosophila* by [28,29], whereby natural polymorphisms influence temperature-sensitive *per* splicing, which determines the phase of the seasonal activity peak [29]. We do not yet know whether northern and southern *per* alleles in *Nasonia* differ in splicing efficiency or posttranslational modification, but our data are consistent with the scenario reported in *Drosophila*. Moreover, the observed, albeit weak, cline in *τ* for virgin females follows that of photoperiodic diapause induction [11,14] and critical photoperiod [8,18]. If *per* participates in photoperiod measurement by fine-tuning critical day length to latitude-dependent requirements, this would suggest an involvement of this clock gene in the photoperiodic timer of *Nasonia.* Consequently, the cline in free running period would reflect a mere “side effect” of the selection pressure on seasonal rhythms. In agreement with this, recent work by [18] found a strong light resetting of the *Nasonia* circadian clock that allows wasps to entrain to a wide range of light–dark cycles, including the northern, more extreme, photoperiods, without negative effect on fitness. 

In conclusion, we described natural variation in the period and phase of daily rhythms between southern and northern *N. vitripennis* lines. Many traits related to circadian activity showed a high level of plasticity, which allows flexibility in daily activities, depending on internal conditions (e.g., mating status) or external environmental conditions (e.g., light–dark cycle, presence of food). Nevertheless, variation between geographic locations was maintained even in the plastic response to different stimuli, suggesting that natural selection acts on the response of the circadian system to the environment and not on the circadian clock, per se. Clearly, more detailed functional experiments are required to reveal the exact molecular mechanism underpinning circadian clock, photoperiodic timer, and their mutual connections.

## Supplementary Materials

The following are available online at www.mdpi.com/xxx/s1, Table S1: Statistical analysis of circadian timing under LD 16:08, Table S2: Statistical analysis of circadian timing under LD 08:16, Figure S1: Correlation between peak of activity and free running period.
