# Supplementary material for: Latitudinal Variation in Circadian Rhythmicity in Nasonia vitripennis
Source: Behav Sci (Basel). 2019 Nov 15;9(11):0. doi: 10.3390/bs9110115 (PMC6912635; doi:10.3390/bs9110115)
Supplement: Supplementary file 1 [file behavsci-09-00115-s001.pdf]

Supplementary Information

Table S1. Timing of onset, peak and offset of activity for Corsica (45°N) (S1, S2, S3, S4, S5) and Oulu (65°N) (N1, N2, N3, N4, N5) wasps under long (LD16:08) and short (LD08:16) day conditions and free running rhythm (τ) under constant condition (DD). ZT (h) is zeitgeber time in hours. Different letters indicate significant differences (*P* < 0.05, ANOVA with a Tukey's post hoc multiple-comparisons test).

	**LD16:08**			**LD08:16**			**DD**	
Lines	Onset ± SE	Peak ± SE	Offset ± SE	Onset ± SE	Peak ± SE	Offset ± SE	τ ± SE	Arrhythmic
	ZT	ZT	ZT	ZT	ZT	ZT	h	%
N1	3.1 ± 0.3	10.8 ± 0.3	18.7 ± 0.6	0.1 ± 0.1	4.5 ± 0.3	11.6 ± 0.4	26.9 ± 0.1	19.4
	(a)	(a)	(a)	(a)	(a)	(ab)	(a)	
N2	1.7 ± 0.4	8.2 ± 0.4	16.2 ± 0.3	23.9 ± 0.2	4.51± 0.2	11.92 ± 0.38	26.7 ± 0.1	55.2
	(ab)	(b)	(b)	(a)	(a)	(b)	(a)	
N3	2.29 ± 0.42	7.2 ± 0.2	17.9 ± 0.2	22.7 ± 0.3	3.15 ± 0.2	10.6 ± 0.3	26.57 ± 0.17	36.7
	(b)	(bc)	(bc)	(ab)	(b)	(a)	(a)	
N4	1.3 ± 0.2	6.7 ± 0.3	14.3 ± 0.3	22.6 ± 0.3	3.12 ± 0.2	10.7 ± 0.4	26.47 ± 0.25	40.7
	(bc)	(bcd)	(c)	(ab)	(b)	(a)	(a)	
N5	1.3 ± 0.5	6.4 ± 0.7	14.6 ± 0.6	22.9 ± 0.2	2.8 ± 0.2	8.7 ± 0.4	26.7 ± 0.1	41.7
	(bc)	(cd)	(bc)	(ab)	(b)	(c)	(a)	
**Overall N**	**2.0** ± **0.2**	**7.9** ± **0.2**	**16.1** ± **0.2**	**23.4** ± **0.2**	**3.5** ± **0.1**	**10.5** ± **0.2**	**26.8** ± **0.1**	**43.5**
S1	1.1 ± 0.4	6.4 ± 0.4	12.7 ± 0.4	21.7 ± 0.2	2.9 ± 0.2	8.7 ± 0.2	23.8 ± 0.1	27.0
	(ab)	(cd)	(c)	(bc)	(b)	(c)	(b)	
S2	0.1 ± 0.1	5.3 ± 0.2	13.1 ± 0.2	22.3 ± 0.2	3.0 ± 0.3	8.6 ± 0.1	24.7 ± 0.2	17.0
	(c)	(d)	(c)	(bc)	(b)	(c)	(c)	
S3	23.8 ± 0.2	5.8 ± 0.3	14.2 ± 0.3	21.1 ± 0.2	1.5 ± 0.2	7.5 ± 0.3	24.4 ± 0.4	51.0
	(c)	(cd)	(c)	(c)	(c)	(c)		
S4	23.9 ± 0.3	5.4 ± 0.4	13.1 ± 0.4	21.9 ± 0.3	3.2 ± 0.3	9.0 ± 0.2	-	100
	(c)	(d)	(c)	(bc)	(b)	(c)	(b)	
S5	23.7 ± 0.2	3.6 ± 0.2	13.2 ± 0.3	21.1 ± 0.2	0.9 ± 0.1	7.8 ± 0.1	24.3 ± 0.1	22.6
	(c)	(e)	(c)	(c)	(c)	(c)	(b)	
**Overall S**	**0.1** ± **0.1**	**5.3** ± **0.2**	**13.2** ± **0.2**	**21.6** ± **0.1**	**2.3** ± **0.1**	**8.3** ± **0.1**	**24.3** ± **0.1**	**38.7**

Table S2: Statistical analysis of circadian timing between southern and northern *Nasonia vitripennis* under LD16:08. Indicated are *P*-values from ANOVA with a Tukey's multiple-comparisons test. In bold $P < 0.05$.

Onset LD16:08

	N1	N2	N3	N4	N5	S1	S2	S3	S4	S5
N1		0.281	0.456	**< 0.001**	**0.006**	**< 0.001**	**< 0.001**	**< 0.001**	**< 0.001**	**< 0.001**
N2			0.948	0.995	0.999	0.938	**0.009**	**< 0.001**	**0.001**	**< 0.001**
N3				0.364	0.601	0.139	**< 0.001**	**< 0.001**	**< 0.001**	**< 0.001**
N4					1.000	0.999	0.161	0.239	**0.040**	**0.010**
N5						0.999	0.277	0.060	0.093	**0.031**
S1							0.337	0.065	0.104	**0.030**
S2								0.997	0.999	0.988
S3									1.000	1.000
S4										0.999
S5										

Peak LD16:08

	N1	N2	N3	N4	N5	S1	S2	S3	S4	S5
N1		**< 0.001**	**< 0.001**	**< 0.001**	**< 0.001**	**< 0.001**	**< 0.001**	**< 0.001**	**< 0.001**	**< 0.001**
N2			0.4334	0.05	**0.01**	**0.008**	**0.009**	**< 0.001**	**0.001**	**< 0.001**
N3				0.989	0.837	0.878	**0.004**	0.138	**0.007**	**< 0.001**
N4					0.999	0.999	0.157	0.791	0.216	**< 0.001**
N5						1.000	0.569	0.988	0.653	**< 0.001**
S1							0.346	0.950	0.435	**< 0.001**
S2								0.994	1.000	**0.009**
S3									0.997	**< 0.001**
S4										**0.010**
S5										

Offset LD16:08

	N1	N2	N3	N4	N5	S1	S2	S3	S4	S5
N1		**< 0.001**	**< 0.001**	**< 0.001**	**< 0.001**	**< 0.001**	**< 0.001**	**< 0.001**	**< 0.001**	**< 0.001**
N2			0.999	**0.031**	0.124	**< 0.001**	**< 0.001**	**0.011**	**< 0.001**	**< 0.001**
N3				0.127	0.366	**< 0.001**	**< 0.001**	0.055	**< 0.001**	**< 0.001**
N4					0.999	0.053	0.272	0.999	0.337	0.463
N5						0.021	0.132	0.999	0.173	0.256
S1							0.998	0.143	0.998	0.986
S2								0.512	1.000	0.999
S3									0.585	0.721
S4										0.999
S5										

Table S3: Statistical analysis of circadian timing between southern and northern *Nasonia vitripennis* under LD08:16. Indicated are *P*-values from ANOVA with a Tukey's multiple-comparisons test. In bold $P < 0.05$.

	Onset LD08:16									
	N1	N2	N3	N4	N5	S1	S2	S3	S4	S5
N1		1.000	0.878	0.611	0.376	**< 0.001**	**0.014**	**< 0.001**	**0.003**	**< 0.001**
N2			0.891	0.60	0.342	**< 0.001**	**0.008**	**< 0.001**	**0.001**	**< 0.001**
N3				0.999	0.999	**0.030**	0.507	**< 0.001**	0.202	**< 0.001**
N4					0.999	0.081	0.751	**< 0.001**	0.382	**< 0.001**
N5						0.157	0.899	**< 0.001**	0.569	**0.001**
S1							0.964	0.765	0.999	0.897
S2								0.093	0.999	0.169
S3									0.401	0.999
S4										0.574
S5										

	Peak LD08:16									
	N1	N2	N3	N4	N5	S1	S2	S3	S4	S5
N1		1.000	**< 0.001**	**< 0.001**	**0.006**	**< 0.001**	**< 0.001**	**< 0.001**	**0.002**	**< 0.001**
N2			**< 0.001**	**< 0.001**	**< 0.001**	**< 0.001**	**0.009**	**< 0.001**	**0.001**	**< 0.001**
N3				1.000	0.999	1.000	1.000	**< 0.001**	0.999	**< 0.001**
N4					0.997	0.999	1.000	**< 0.001**	0.999	0.999
N5						0.999	0.999	**0.001**	0.985	**< 0.001**
S1							1.000	**< 0.001**	0.999	**< 0.001**
S2								**< 0.001**	0.999	**< 0.001**
S3									**< 0.001**	0.709
S4										**< 0.001**
S5										

	Offset LD08:16									
	N1	N2	N3	N4	N5	S1	S2	S3	S4	S5
N1		0.999	0.156	0.369	**< 0.001**	**< 0.001**	**< 0.001**	**< 0.001**	**< 0.001**	**< 0.001**
N2			**0.008**	**0.04**	**< 0.001**	**< 0.001**	**< 0.001**	**< 0.001**	**< 0.001**	**< 0.001**
N3				0.999	**0.016**	**0.010**	**0.006**	**< 0.001**	0.171	**< 0.001**
N4					**0.006**	**0.004**	**0.002**	**< 0.001**	**0.079**	**< 0.001**
N5						1.000	1.000	0.316	0.999	0.697
S1							1.000	0.292	0.998	0.677
S2								0.413	0.994	0.799
S3									0.053	0.999
S4										0.211
S5										
